# Reinterpretation of common pathogenic variants in ClinVar revealed a high proportion of downgrades

**DOI:** 10.1038/s41598-019-57335-5

**Published:** 2020-01-15

**Authors:** Jiale Xiang, Jiyun Yang, Lisha Chen, Qiang Chen, Haiyan Yang, Chengcheng Sun, Qing Zhou, Zhiyu Peng

**Affiliations:** 1 BGI Genomics, BGI-Shenzhen, Shenzhen, 518083 China; 2 School of Medicine, University of Electronic Science and Technology of China, Chengdu, 610072 China; 3 Sichuan Provincial Key Laboratory for Human Disease Gene Study, Hospital of the University of Electronic Science and Technology of China and Sichuan Provincial People’s Hospital, Chengdu, 610072 China; 4 BGI Education Center, University of Chinese Academy of Sciences, Shenzhen, 518083 China; 5 Department of Stomatology, The Third Affiliated Hospital of Sun Yat-sen University, Guangzhou, 510630 China; 6 BGI College, Zhengzhou University, Zhengzhou, 45000 China; 7 School of Basic Medicine, Qingdao University, 308 Ningxia Road, Qingdao, 266071 China; 8 The MOE Key Laboratory of Biosystems Homeostasis & Protection, Life Sciences Institute, Zhejiang University, Hangzhou, 310058 China

**Keywords:** Molecular medicine, Diseases

## Abstract

High-frequency disease-causing alleles exist, but their number is rather small. This study aimed to interpret and reclassify common pathogenic (P) and likely pathogenic (LP) variants in ClinVar and to identify indicators linked with reclassification. We analyzed P/LP variants without conflicting interpretations in ClinVar. Only variants with an allele frequency exceeding 0.5% in at least one ancestry in gnomAD were included. Variants were manually interpreted according to the guidelines of the American College of Medical Genetics and Genomics and the Association for Molecular Pathology. Of 326 variants retrieved, 217 variants in 173 genes were selected for curation. Overall, 87 (40%) variants were downgraded to benign, likely benign or variant of uncertain significance. Five variants (2%) were found to be more likely to be risk factors. Most of the reclassifications were of variants with a low rank, an older classification, a higher allele frequency, or which were collected through methods other than clinical testing. ClinVar provides a universal platform for users who intend to share the classification variants, resulting in the improved concordance of variant interpretation. P/LP variants with a high allele frequency should be used with caution. Ongoing improvements would further improve the practicability of ClinVar database.

## Introduction

ClinVar is a freely available, public archive of interpretations of clinically relevant variants which has received an increasing number of submissions globally^1^. It provides a site for data sharing among researchers, laboratories, expert groups, and patients, improving the accuracy of variant interpretation^2^. To date, over 500,000 variants have been submitted to ClinVar^1^. However, the database also includes misclassified variants^3^, which are challenging to detect among the hundreds of thousands of variants in the record.

Disease-causing variants are generally rare. High-frequency alleles (i.e. >0.5%) exist but their number is rather small. The accurate interpretation of these variants has a significant impact in clinical and research settings. For example, their pathogenicity affects the gene-specific allele frequency thresholds used as evidence for pathogenic or benign variant interpretation^4^. Recently, ClinGen experts curated 103 variants with an allele frequency exceeding 5% and concluded that only four of them were pathogenic^5^. Whiffin *et al*. curated 43 variants classified in ClinVar as pathogenic (P)/likely pathogenic (LP) that were insufficiently rare in at least one ExAC population and found that 42 of them should be reclassified as variant of uncertain significance (VUS)^6^.

In this study, we focused on P and LP variants found in ClinVar without conflicting interpretations and which were common, defined here as having an allele frequency greater than 0.5% in at least one ancestry in The Genome Aggregation Database (gnomAD)^7^. We attempted to assess and reclassify variants that are compatible with the American College of Medical Genetics and Genomics and the Association for Molecular Pathology (ACMG/AMP) criteria^8^, to identify indicators for the reclassification, and to analyze the reasons for downgraded classifications. Our results will be of interest to ClinVar users, and they underline the need for ongoing improvements and continuous data sharing in variant interpretation.

## Methods

ClinVar P or LP variants without conflicting interpretations were extracted from raw VCF files that were downloaded on 1 March 2019. Allele frequencies of variants were retrieved from gnomAD v2.1^7^ on 31 December 2018 and were applied when they had a minimum 2,000 alleles^5^. Then, the ClinVar P/LP variants were matched with the gnomAD variants to identify ClinVar variants present in gnomAD.

Variants were excluded from this study if: (1) the allele frequency was lower than 0.5% after filtering out the non-pass calls in gnomAD; (2) the P/LP classification was “risk factors”, “protective”, or “other”, which are not considered pathogenic; (3) the variant was somatic; (4) the variant was in a gene with an OMIM phenotype of “susceptibility to complex disease or infection”, “Non-diseases”, “provisional phenotype-gene relationship” or “Not included” (https://www.omim.org/help/faq), since these variants were not compatible with ACMG/AMP criteria^8^; (5) the variant was curated by expert panels or included in guidelines (three or four colored stars in ClinVar).

The remaining variants were each assigned to two biocurators. Each biocurator interpreted the variants independently using the ACMG/AMP standards and guidelines^8^. Interpreting discordances (n = 36) were first discussed between the two biocurators themselves. Then, the 36 classifications and the ACMG/AMP criteria applied were discussed with Dr. Zhou and Dr. Peng by phone conferences in twice. The final interpretation results were subsequently reviewed and verified by Dr. Zhou and Dr. Peng.

To elucidate the characteristics of the common P/LP variants and explore their reclassification, we stratified them by review status, last date evaluated, allele frequency, and collection method. The allele frequency was retrieved from gnomAD, and all of the other information was collected from ClinVar. The review status was depicted by colored stars, which were ranked according to the source and level of review for each submitted variant assertion^2^.

As the scope of this study was to determine medically actionable reclassifications, we grouped P and LP variants as P/LP, and benign (B) and likely benign (LB) classification as B/LB. We used chi-squared tests for between-group comparisons. Statistical analysis was performed with IBM SPSS Statistics, version 24 (SPSS). A *p* value of less than 0.05 in two-tailed tests was considered statistically significant. As the study investigated publicly available data, it was exempt from the need for approval by the Institutional Review Board of BGI.

## Results

**Figure 1:**
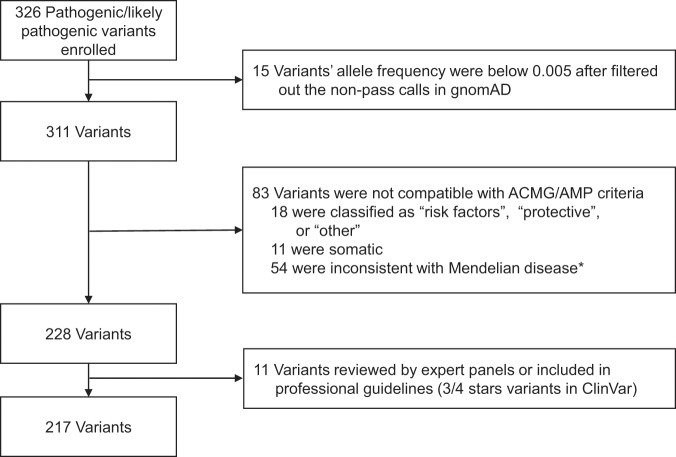
The enrollment of ClinVar pathogenic and likely pathogenic variants for curation. *Variants in genes with their phenotype in OMIM were “susceptibility to complex disease or infection”, “Non-diseases”, “provisional phenotype-gene relationship” or “Not included”.

In total, 326 P/LP variants without conflicting annotations and with an allele frequency exceeding 0.5% in at least one gnomAD ancestry were retrieved from ClinVar. We excluded 109 variants from further analysis, 76% (83/109) of which were ruled out because they were not compatible with ACMG/AMP variant interpretation criteria (Supplementary Table S1) and 11 of which had been curated by expert panels or included in professional guidelines. This left 217 variants to be assigned to biocurators (Fig. 1).

Of the 217 P/LP variants, 87 (40%) were downgraded to B/LB/VUS. Four variants (2%) were found to be more likely to be risk factors (Table 1). 12% (27/217) of the curated variants had an allele frequency greater than 5% in at least one ancestry in gnomAD. Of those, 96% (26/27) were downgraded to B/LB/VUS. The remaining variant was NM_001168357.1(*PLA2G7*):c.835 G > T (p.Val279Phe), which had an allele frequency of 5.6% in East Asian and was classified as a risk factor for asthma^9^ and cardiovascular disease^10^. Variants with an allele frequency between 0.005 and 0.01 had fewer downgrades than those between 0.01 and 0.05 (23% vs. 44%, *p* < 0.01).

**Table 1:** Reclassification outcomes of ClinVar P//LP variants with an allele frequency greater than 0.005^a^.

Reclassification, n (%)	B/LB/VUS			P/LP	Risk factor	All
Characteristic	B/LB	VUS	Total			
All	46 (21)	41 (19)	87 (40)	126 (58)	5 (2)	217 (100)
Maximal allele frequency						
[0.005. 0.01)	2 (2)	22 (21)	24 (23)	80 (76)	1 (1)	105 (100)
[0.01, 0.05)	19 (22)	18 (21)	37 (44)	45 (53)	3 (4)	85 (100)
[0.05, 1)	25 (93)	1 (4)	26 (96)	0 (0)	1 (4)	27 (100)
Collection method						
Clinical testing	11 (9)	9 (8)	20 (17)	95 (81)	2 (2)	117 (100)
Literature only	22 (29)	26 (34)	48 (63)	25 (33)	3 (4)	76 (100)
Research	8 (57)	4 (29)	12 (86)	2 (14)	0 (0)	14 (100)
Reference population	0 (0)	2 (40)	2 (40)	3 (60)	0 (0)	5 (100)
Case-control	2 (100)	0 (0)	2 (100)	0 (0)	0 (0)	2 (100)
Not provided	3 (100)	0 (0)	3 (100)	0 (0)	0 (0)	3 (100)
Last evaluated (year)						
2014 and earlier	17 (28)	23 (38)	40 (67)	18 (30)	2 (3)	60 (100)
2015–2019	19 (13)	14 (10)	33 (23)	107 (75)	3 (2)	143 (100)
Unspecified	10 (71)	4 (29)	14 (100)	0 (0)	0 (0)	14 (100)
Review status						
0 star	42 (41)	30 (29)	72 (71)	27 (26)	3 (3)	102 (100)
1 star	4 (8)	11 (22)	15 (31)	34 (69)	0 (0)	49 (100)
2 stars	0 (0)	0 (0)	0 (0)	64 (97)	2 (3)	66 (100)

^a^Percentages may not sum to 100 because of rounding.

Abbreviations: P: Pathogenic; LP: Likely pathogenic; VUS: Variant of uncertain significance; LB: Likely benign; B: Benign.

Analyzing the variants in terms of collection method revealed that variants collected from clinical testing had a lower probability of being downgraded (17%) compared with those collected from the literature only (63%) or from research (86%); these differences were statistically significant (*p* < 0.001). Moreover, it is evident that the older classifications were more likely to be reclassified than those curated after 2014 (67% vs. 23%, *p* < 0.001). Interestingly, 28% (60/217) of variants had not been updated since the structured ACMG/AMP variant interpretation guidelines were proposed in 2015.

A large number of variants (47%; 102/217) were submitted with neither an assertion nor a documented method provided, which was depicted with zero stars in ClinVar^2^. We found that 71% (72/102) of these should be downgraded, a significantly higher proportion than was found for variants with criteria provided (one-star variant: 31%, 15/49). No medically actionable downgrade was observed in variants with two stars, but two variants (NM_000055.2(*BCHE*):c.293 A > G and NM_000506.3(*F2*)c.*97 G > A) proved more likely to be a risk factor or drug response. NM_000055.2(*BCHE*):c.293 A > G causes Butyrylcholinesterase deficiency (OMIM #617936) and affected individuals are asymptomatic unless exposed to neuromuscular blocking agents^11^. NM_000506.3(*F2*)c.*97 G > A was associated with myocardial infarction^12^ and recurrent venous thromboembolism^13^.

Of 87 B/LP/VUS variants and 5 risk factors (92 in total), we attempted to analyze the reason for downgrade. 65% (60/92) of the variants were downgraded because the evidence from relevant publications were not sufficient to support their pathogenicity. Of note, 10% (9/92) variants were correctly interpreted in the publication (polymorphism or risk-factor), but incorrectly submitted (pathogenic or likely pathogenic) to ClinVar. The reason for the remaining 25% was undetermined because no citation in ClinVar or the variant was not found in the citation (Column N in the Supplementary Table S1).

## Discussion

It is currently well recognized that variants thought to be disease-causing are often subsequently downgraded^14^. Researchers have noted that it is better for variant classifications to be uncertain than to be wrong, and ideally false calls of pathogenicity should be avoided in the first place^15^. Here, we review high-frequency P/LP variants in ClinVar and find that a high proportion (40%) should be downgraded. The proportion remained high (24%) even when we narrowed our analysis to variants that were evaluated after 2014, when population databases became publicly available, highlighting the need for stringent evaluation of the clinical significance of variants in clinical practice.

P/LP variants with an allele frequency greater than 0.5% have important roles in both clinical and research settings. Given their high frequency in the general population, they are more easily identified than rare alleles but are harder to interpret in some circumstances due to concerns about the founder effect. Therefore, the high frequency of these alleles does not rule out their pathogenicity^5^, which must be determined by more rigorous reviews or even expert consensus^16^. Accurate classification of such variants determines the disorder-specific allele frequency thresholds and ultimately affects the classification of other variants^4^. Moreover, the high frequency of one variant may exceed the cumulative frequency of all other rare P/LP variants. Inclusion or exclusion of these variants substantially influences the risk assessment of genetic conditions^17^, which may ultimately affect panel design for expanded carrier screening.

A high frequency of disease-causing variants might be explained in several ways. First, the founder effect and heterozygote advantage could result in an unusually high frequency in a specific population^18^. Second, deleterious mutations are not fully penetrant^19^, facilitating their inheritance in evolution. Third, some variants are risk factors which only predispose affected individuals to non-lethal disorders. For instance, one curated variant, NM_000055.2(*BCHE*):c.293 A > G, is a risk factor and affected individuals are asymptomatic unless exposed to neuromuscular blocking agents^11^. Finally, public database users should be aware that pseudogene homology might inflate the allele frequency in public genome/exome databases. For example, one of variants filtered out in this study, NM_002769.4(*PRSS1*):c.86 A > T, is one of the leading causes for hereditary pancreatitis. Its overall allele frequency was 28.3% and was greater than 20% in each ancestry in gnomAD, contrary to clinical observations of disease prevalence. This discrepancy resulted from a mapping error due to the existence of a highly paralogous region in *PRSS2*.

We found that several other indicators besides allele frequency were also associated with incorrectly ascertained variants. Specifically, a previous study focused on variants classified by two or more submitters in ClinVar and found that newer classifications and variants identified by clinical testing had greater concordance than older classifications and those collected by other methods^20^. Similarly, although we only focused on P/LP variants without discordance in this study, our findings demonstrated that clinical testing methods and newer classifications had fewer downgrades. Furthermore, consistent with a recent study by Shah *et al*.^3^, we also concluded that lower ranked variants (based on the number of colored stars) were more likely to be reclassified.

This study has two limitations. First, biocurators are unlikely to have comprehensive knowledge of all conditions related to 217 variants in 173 genes. The lack of disease-specific knowledge may sometimes lead to an inappropriate interpretation of certain criteria. To minimize this, we assigned the variants to two independent biocurators and discussed any discordance with senior researchers. Second, our interpretation of the variants was based only on publicly available evidence from the literature. However, submitters may have an in-house database or unpublished evidence to support the pathogenicity of variants they submitted^21^. In this case, the downgrade may be over-inflated. This reinforces the importance of data sharing in the scientific community.

At the moment, ClinVar recommends fourteen categories for records of clinical significance (https://www.ncbi.nlm.nih.gov/clinvar/docs/clinsig/). Variants with low penetrance are recommended to be submitted as “Pathogenic” with information about the penetrance included in a “Comment on clinical significance”. However, during our investigation we observed that penetrance information is generally lacking. Moreover, low penetrance variants had particularly high discordance among different submitters^20^. We recommend that a more specific terminology for low penetrance variants should be developed, which would significantly improve the practicability of ClinVar database.

In conclusion, ClinVar provides a universal platform for users who intend to share the classification of the clinical significance of variants, resulting in the improved concordance of variant interpretation. In practice, variants with older classifications, lower ranks or unexpectedly high allele frequency should be interpreted with caution. Ongoing improvements by ClinVar managers to refine the classification system may help alleviate the problem.

## Supplementary information

Dataset 1.

## References

[1] Landrum MJ (2018). ClinVar: improving access to variant interpretations and supporting evidence. Nucleic Acids Res..

[2] Rehm HL (2015). ClinGen–the Clinical Genome Resource. N. Engl. J. Med..

[3] Shah N (2018). Identification of Misclassified ClinVar Variants via Disease Population Prevalence. Am. J. Hum. Genet..

[4] Zastrow DB (2018). Unique aspects of sequence variant interpretation for inborn errors of metabolism (IEM): The ClinGen IEM Working Group and the Phenylalanine Hydroxylase Gene. Hum. Mutat..

[5] Ghosh R (2018). Updated recommendation for the benign stand-alone ACMG/AMP criterion. Hum. Mutat..

[6] Whiffin N (2017). Using high-resolution variant frequencies to empower clinical genome interpretation. Genet. Med..

[7] Lek M (2016). Analysis of protein-coding genetic variation in 60,706 humans. Nature.

[8] Richards S (2015). Standards and guidelines for the interpretation of sequence variants: a joint consensus recommendation of the American College of Medical Genetics and Genomics and the Association for Molecular Pathology. Genet. Med..

[9] Stafforini DM (1996). Platelet-activating factor acetylhydrolase deficiency. A missense mutation near the active site of an anti-inflammatory phospholipase. J. Clin. Invest..

[10] Jang Y (2006). The Val279Phe variant of the lipoprotein-associated phospholipase A2 gene is associated with catalytic activities and cardiovascular disease in Korean men. J. Clin. Endocrinol. Metab..

[11] Lockridge O (2015). Review of human butyrylcholinesterase structure, function, genetic variants, history of use in the clinic, and potential therapeutic uses. Pharmacol. Ther..

[12] Rosendaal FR (1997). A common prothrombin variant (20210 G to A) increases the risk of myocardial infarction in young women. Blood.

[13] De Stefano V (2001). The risk of recurrent venous thromboembolism among heterozygous carriers of the G20210A prothrombin gene mutation. Br. J. Haematol..

[14] Manrai AK, Ioannidis JP, Kohane IS (2016). Clinical Genomics: From Pathogenicity Claims to Quantitative Risk Estimates. JAMA.

[15] Weck KE (2018). Interpretation of genomic sequencing: variants should be considered uncertain until proven guilty. Genet. Med..

[16] Shen J (2019). Consensus interpretation of the p.Met34Thr and p.Val37Ile variants in GJB2 by the ClinGen Hearing Loss Expert Panel. Genet. Med..

[17] Guo MH, Gregg AR (2019). Estimating yields of prenatal carrier screening and implications for design of expanded carrier screening panels. Genet. Med..

[18] Risch N (2001). Molecular epidemiology of Tay-Sachs disease. Adv. Genet..

[19] Wright CF (2019). Assessing the Pathogenicity, Penetrance, and Expressivity of Putative Disease-Causing Variants in a Population Setting. Am. J. Hum. Genet..

[20] Yang S (2017). Sources of discordance among germ-line variant classifications in ClinVar. Genet. Med..

[21] DiStefano MT (2019). ClinGen expert clinical validity curation of 164 hearing loss gene-disease pairs. Genet. Med..

