# Author Correction: Reinterpretation of common pathogenic variants in ClinVar revealed a high proportion of downgrades

**DOI:** 10.1038/s41598-020-67915-5

**Published:** 2020-07-09

**Authors:** Jiale Xiang, Jiyun Yang, Lisha Chen, Qiang Chen, Haiyan Yang, Chengcheng Sun, Qing Zhou, Zhiyu Peng

**Affiliations:** 1 BGI Genomics, BGI-Shenzhen, Shenzhen, 518083 China; 2 School of Medicine, University of Electronic Science and Technology of China, Chengdu, 610072 China; 3 Sichuan Provincial Key Laboratory for Human Disease Gene Study, Hospital of the University of Electronic Science and Technology of China and Sichuan Provincial People’s Hospital, Chengdu, 610072 China; 4 BGI Education Center, University of Chinese Academy of Sciences, Shenzhen, 518083 China; 5 Department of Stomatology, The Third Affiliated Hospital of Sun Yat-Sen University, Guangzhou, 510630 China; 6 BGI College, Zhengzhou University, Zhengzhou, 45000 China; 7 School of Basic Medicine, Qingdao University, 308 Ningxia Road, Qingdao, 266071 China; 8 The MOE Key Laboratory of Biosystems Homeostasis & Protection, Life Sciences Institute, Zhejiang University, Hangzhou, 310058 China

Correction to: *Scientific Reports* 10.1038/s41598-019-57335-5, published online 15 January 2020

This Article contains errors in the Results section where,

“Four variants (2%) were found to be more likely to be risk factors (Table 1).”

Should read:

“Five variants (2%) were found to be more likely to be risk factors (Table 1).”

Additionally, there is an error in Table 1 where the P/LP value for the Characteristic All is incorrect.

The correct value should be 125 (58).

